# Clinical Data Mining of Phenotypic Network in Angina Pectoris of Coronary Heart Disease

**DOI:** 10.1155/2012/546230

**Published:** 2012-06-12

**Authors:** Jianxin Chen, Peng Lu, Xiaohan Zuo, Qi Shi, Huihui Zhao, Liangtao Luo, Jianqiang Yi, Chenglong Zheng, Yi Yang, Wei Wang

**Affiliations:** ^1^Beijing University of Chinese Medicine, 11 Bei San Huan Dong Lu, ChaoYang District, Beijing 100029, China; ^2^Institute of Automation, Chinese Academy of Sciences, 95 Dong Lu, Zhong-guan-cun, Hai Dian District, Beijing 100190, China

## Abstract

Coronary heart disease (CHD) is the leading causes of morbidity and mortality in China. The diagnosis of CHD in Traditional Chinese Medicine (TCM) was mainly based on experience in the past. In this paper, we proposed four MI-based association algorithms to analyze phenotype networks of CHD, and established scale of syndromes to automatically generate the diagnosis of patients based on their phenotypes. We also compared the change of core syndromes that CHD were combined with other diseases, and presented the different phenotype spectra.

## 1. Introduction

Coronary heart disease (CHD) is the leading causes of morbidity and mortality in China [1].

Angina pectoris (AP) is one of the most common types of CHD. Its treatment in modern medicine mainly includes nitrates, *β*-blockers, Ca^2+^ channel blockers, and coronary angioplasty or coronary artery bypass graft surgery. However, its side effects could be ignored. Traditional Chinese Medicine (TCM) presented a complementary and alternative avenue to treating AP of CHD. It uses a holistic concept to balance whole body, not like western medicine whose treatment of AP places heavily on healing of the heart organ.

TCM has a history of more than 1000 years to fight with CHD. The Chinese ancients used words “thoracic obstruction (Xiongbi in Mandarin)” to describe phenotypes of CHD and piled thousands of formula to treat CHD. The key concept of TCM is syndrome, which is the core of TCM diagnosis and therapy theory. A syndrome is composed of a set of phenotypes, Wu et al. [2] proposed a computational framework called CIPHER that integrates information from phenotypes and genes, and the preferable results confirmed the biological significance of phenotypes. Li et al. [3] investigated the key pathological principle, ZHENG, in the context of the neuroendocrine immune (NEI) system and reported their important finding about predominant parts in the Cold/Hot ZHENG network, the connections between these two networks, and interaction pathways the genes related to ZHENG-related diseases were mainly present in. All of these were subsequently verified by experiments on a rate model of collagen-induced arthritis. Their excellent work demonstrated the thousand-year-old concept of ZHENG might have a molecular basis with NEI as background for the first time.

The past decades of CHD syndrome-related research effort place heavily on blood stasis syndrome (BSS). Most of them were to investigate biological basis of blood stasis syndrome in the context of CHD, for example, proteomic study of BSS [4], animal model establishment of BSS in the context of myocardial infarction [5], the association between BSS and clinical biological index [6], or the action mechanism of formula on treating BSS [7]. Despite these progresses made in complementary and alternative research of CHD, the standardization and modernization of syndromes in the context of CHD are still far from need of worldwide clinical applications. The correct diagnosis of syndromes in the context of CHD plays a key role in modernization of syndromes. However, due to complex pathopoiesis factors of CHD and relatively simple statistical data analysis methods, a diagnostic scale of syndromes in CHD was hard to establish.

Traditionally, a syndrome scale was build according to three steps. The first was to determine phenotype pool of the syndrome. Then, the score or weight of each phenotype was computed. The final step was to determine a diagnostic threshold of the syndrome. Among these, the first step is most important. Till now, the most used method to determine phenotype pool was subjective, for example, by using TCM experts' questionnaire, which is hard to enhance diagnosis accuracy of syndromes. The complex data analysis methods for establishing diagnostic scale of syndromes were urgent.

In this paper, we presented mutual-information- (MI-) based complex system computational methods to objectively determine phenotype pools of syndromes. We carried out a large sample cohort of CHD subjects. Four MI-based association algorithms were compared to retrieve phenotype pairs with significant association. The phenotype networks were established accordingly. A validation algorithm was presented to choose a better algorithm, and thus phenotype pool of each syndrome in the context of CHD was determined. We also investigate different phenotype spectra of CHD when combined with hypertension, diabetes, hyperlipemia, and chronic heart failure.

## 2. Materials and Methods

### 2.1. AP of CHD Cohort

2050 AP subjects aged between 45 and 75 were collected from 7 clinical centers located in 7 provinces in China from the same demographic area and at the same time from November 2008 to November 2010. Stable AP was strictly diagnosed according to *ACC/AHA/ACP-ASIM Guidelines for the Management of Patients with Chronic Stable Angina* [8]. Unstable AP was diagnosed as per *Diagnosis and Treatment Recommendation of Unstable Angina Pectoris* published by Chinese Society of Cardiology [8]. The exclusion criteria were composed of four conditions. (1) Patients with acute myocardial infarction, myocarditis, pericardial disease, cardiac neurosis, intercostal neuralgia, menopausal syndrome, and severe chest pain caused by cervical spondylosis were excluded; (2) patients with AP caused by other diseases such as rheumatic fever, syphilis, congenital coronary abnormalities, hypertrophic cardiomyopathy, aortic stenosis, or regurgitation were excluded; (3) patients with combined diseases such as stroke, pulmonary infection, nephritis, renal failure, urinary tract infections, rheumatism, severe arrhythmia, cancer, liver, kidney, hematopoietic system, primary and other serious diseases, uncontrolled hypertension or systolic blood pressure *⩾*180 mmHg or diastolic blood pressure *⩾*110 mmHg after blood pressure control were also excluded; (4) pregnancy or breast-feeding women, patients with allergy (included in the state except when the nonallergic), or the mentally ill were excluded from the cohort.

The study protocol was approved by both the ethics committee of Dongzhimen Hospital affiliated to Beijing University of Chinese Medicine and the local ethics committee of the collaborative hospitals. All subjects who included in the study provided written informed consent.

### 2.2. Phenotype Information Determination and Collection

Besides demographic information, characteristics of disease history, medication information, as well as main symptoms and signs in western medicine, 107 phenotypic variables composed of symptoms, signs, tongue, and pulse information were also carefully investigated. They were collected by watching, listening, inquiring, and pulse feeling. The inclusion of the 107 variables was determined by a combination of three avenues. Firstly, literatures with AP and Traditional Chinese Medicine were fully collected from publicly accessed databases. All phenotypic variables were manually acquired from the literatures. Synonym and phenotype with similar clinical meaning were combined, forming a candidate pool of TCM phenotype terms for AP of CHD. Alternatively, two rounds of TCM experts questionnaire were carried out to screen a compact set of phenotype variables based on an idea that clinical experts consensus on the phenotype information of diseases could reduce the complexity of phenotype and increase the objectivity of the determination of phenotype to be clinically investigated. Finally, a preliminary clinical epidemiology of 100 AP cases was performed to investigate frequency of each phenotype. A cut of 5% was used to determine a final version of phenotypes of AP.

### 2.3. Data Analysis

Frequency of each phenotype was computed and descending ranked. Association between phenotypes was calculated by revised mutual information [9]. Four computational algorithms were used or presented to retrieve several numbers of associations to construct phenotype network for AP. A validation strategy was presented to evaluate each network and screen a better algorithm for building such network. The subnetwork of AP combined with hypertension, diabetes hyperlipemia, or chronic heart failure was constructed, respectively. The difference between each subnetwork was significantly understood to investigate phenotype spectra of AP when combined with distinctive diseases. Pajek 2.0 was used to build complex phenotype networks [10].

## 3. Results and Discussion

### 3.1. Basic Statistics

**Figure 1:**
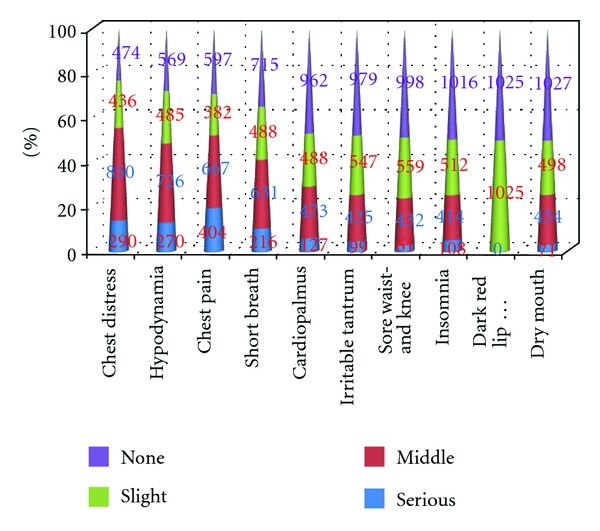
The initial 10 phenotypes and their frequencies of four classes, that is, serious, middle, slight, and none. Eight phenotypes occurred at more than 50% of subjects.

Table 1 listed the basic information of demography and combined diseases of the study cohort. The average age of the AP subjects was 62.95 ± 10.56. Hypertension occupied more than 67% of AP cohort, indicating that it is a key risk factor to AP by the retrospective epidemiology. Nearly two in three AP patients are male. As shown in Figure 1, eight phenotypes appeared in more than 50% subjects. The most frequent phenotype in AP subject was chest distress, which is a typical symptom of AP. It is surprising that hypodynamia is with slightly higher frequency than chest pain. The latter is an anther typical phenotype following with AP. However, this situation is solvable by mean of viewpoint of TCM. Hypodynamia is a characteristic symptom of Qi deficiency syndrome in TCM, which is considered as key pathology of AP.

Mutual information is good at quantitatively describing association between categorical variables. As depicted in Table 2, the top 10 phenotype pair and their association were given. A phenotype with an asterisk in the right cornu superius means that it is in the list of top 10 phenotypes of AP. It is found that phenotype with high-frequency phenotype was prone to associated with the other high-frequency phenotype. However, they only occupied 50% of top 10 phenotype pairs with highest MI, which indicated that MI could balance between frequency and association. A phenotype pair with high MI association not only showed a high value of cooccurrence but also described a high frequency of co-nonoccurrence. The latter usually makes two totally adverse and useless phenotypes highly associated (data not shown here). Thus, the revised MI was used here to prevent negative association from positive association pairs.

The inherent drawback of MI algorithm is that it ignores frequency of the features, so it is inclined to select lower-frequency features such as co-nonoccurrence phenotype pairs. For this reason, we proposed a revised MI that takes use the “positive occurrence frequency” to control the growth of co-nonoccurrence pairs in MI computation. The positive occurrence frequency is defined as the frequency of cooccurrence of phenotype pairs. The positive occurrence frequency of strong correlation phenotypes is bigger (close to 1), and, in theory, the positive occurrence frequency of adverse phenotypes should be 0, for that it is impossible for one patient to get two adverse phenotypes at the same time. So we redefine the MI as

(1) $$\begin{array}{cc} & \Delta \mu \left(X_{i},X_{j}\right)\\ & \;=\{\begin{array}{c} H\left(X_{i}\right)+H\left(X_{j}\right)-H\left(X_{i},X_{j}\right),\;\text{Po}\left(i,j\right)\geq \delta ,\\ H\left(X_{i}\right)+H\left(X_{j}\right)-b^{*}H\left(X_{i},X_{j}\right),\;\text{Po}\left(i,j\right)\geq \delta , \end{array} \end{array}$$

where Po(*i*, *j*) is the positive occurrence frequency of feature *i* and *j*, *δ* is preassigned positive quantity, we call it POF threshold in this paper. When *δ* = 0, the revised version of MI is the traditional form of MI, so the revised MI is an extended version of traditional MI. *b* is a real number and is greater than 1, it can be seen as a penalty coefficient.

It is this better merit of MI that its four extensions would be used to establish phenotype network of AP and to further investigate the association between subnetworks and syndrome in TCM.

### 3.2. Complex Phenotype Network

The four MI-based algorithms only presented information on various computational methods of associations between phenotypes. Significant association algorithm was defined to determine number of associated phenotypes where the network was established. A phenotype pair that composed of *P*_*A*_ and *P*_*B*_ was defined as significant association as follows: *P*_*A*_ ∈ *R*(*P*_*B*_) and *P*_*B*_ ∈ *R*(*P*_*A*_). Where *R*(*P*_*A*_) and *R*(*P*_*B*_) denoted the top *N* associated phenotypes of the phenotype *P*_*A*_ and *P*_*B*_, respectively. The number *N* was determined by presenting a concept of information utilization, which was defined as ratio of maximal number of phenotypes in discovered pattern to *N*. Here, *N* = 6 was found to achieve a high information utilization with 83.33% (equal to 5/6). 107 phenotypes were retrieved their *R*(*P*_*i*_, *i* = 1,2,…, 107) according to revised MI, respectively, resulting a number of 120 significant association pairs were computed. The other three MI-based algorithms were presented as follows.

1. Revised MI-based association of a phenotype pair [8].
2. Revised MI divided by between-phenotype distance [11]. The between-phenotype distance was defined as
  (2) $$d\left(x,y\right)=\frac{\sum_{i=1}^{2050}I\left(x,y,i\right)\left|B\left(x,i\right)-B\left(y,i\right)\right|}{\sum_{i=1}^{2050}I\left(x,y,i\right)},$$
  where *I*(*x*, *y*, *i*) = 1 means phenotype *x* and phenotype *y* simultaneously appeared on the *i*th subject and = 0 otherwise. *B*(*x*, *i*) is denoted for the none (0), slight (1), middle (2), and serious (3) of phenotype *x*.
3. Revised MI divided by Euclidean distance between phenotype pair.

**Figure 2:**
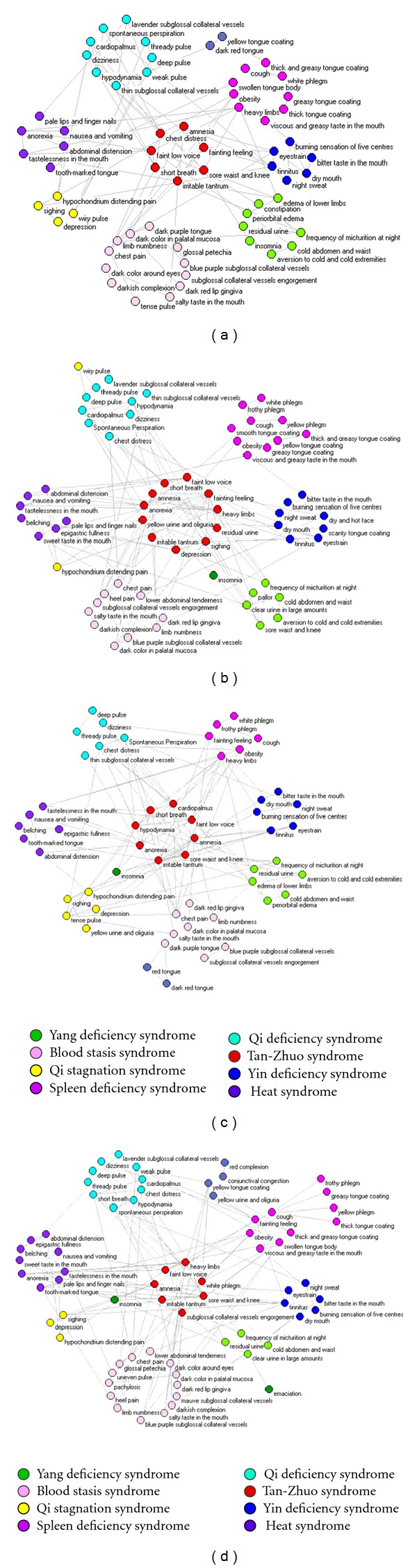
The phenotype networks for AP built by the four MI-based algorithms.

107 phenotypes were observed and collected from clinical data under the strict quality control. In this process, there was no intervention of subjective factors. It was objective descriptions of patients' symptoms. Mutual information (MI) from complex system was used to describe association between phenotypes. The association data was consolidated into adjacency matrix and then converted into the format that Pajek software required. Pajek software 2.0 was used to analyze the node degrees of the phenotypes. With the command of “Layout-Energy-Kamada-Kawai-Separate Components,” we drew the phenotype networks according to different colors and different degrees. The principles of network adjustment were delete the isolated nodes, mediate positions of other nodes with manual operation. Nodes and edges of the network could not be deleted. Then, we exported the network figures in Bitmap format. In Figure 2, the phenotypes networks were made up of the centre network (red colors) and the surrounding networks with different colors. In Figures 2(a) to 2(d), networks with the same colors reflected the same syndromes. For example, a combination of eyestrain, tinnitus, night sweat, dry mouth, bitter taste in the mouse, and burning sensation of five centres means Yin deficiency according to TCM theory (Figure 2(a)). By using this clue, the four networks involved seven syndromes, that is, Qi deficiency syndrome, Yin deficiency syndrome, Yang deficiency syndrome, Spleen deficiency syndrome, Blood stasis syndrome, Tan-Zhuo syndrome, Qi stagnation syndrome. What is more, there were two other cases needed to be explained. Firstly, the numbers of nodes that reflected “heart syndrome” were small, and these nodes were not in the presence of all the phenotypes networks. So the heat syndrome was not classified as the main syndromes. Secondly, emaciation and insomnia were not the specific responses of syndromes in clinical process. There two phenotypes may appear in patients with different syndromes. We therefore denoted them with another color. In order to express more clearly, we had already added the legend in the revised paper.

To quantitatively confirm this finding, we took the proportion of edges between nodes from different classes (colored subnets) as a measure of the efficiency of clustering. For comparison, we generated 100 randomized networks by randomly shuffling the edges between nodes while keeping the number of edges and nodes unchanged, and we find that the actual proportion of the “between classes edges” is significantly smaller than the average ones (*P* < 10^−40^). Actually, the *P* values of the four networks in Figure 2 are 6.47*E* − 130, 5.89*E* − 102, 1.74*E* − 119, 2.99*E* − 41 under 100 randomized networks, and when we expand the number of networks to 1000, the *P* values reduced to 0. This result confirms the fact that nodes in the networks are intended to cluster into subnetworks as we declared.

Indeed, the unsupervised clustering of phenotypes here coincide the concept of complementary and alternative medicine and a subnetwork is responsible for a syndrome in TCM. For example, a combination of chest distress, faint low voice, amnesia, short breath, fainting feeling, sore waist and knee, and irritable tantrum means Qi deficiency according to TCM theory. By using this clue, the four networks involved seven syndromes, that is, Qi deficiency syndrome, Yin deficiency syndrome, Yang deficiency syndrome, spleen deficiency syndrome, blood stasis syndrome, Tan-Zhuo syndrome, Qi stagnation syndrome. The four algorithms involved 44, 54, 64, and 69 phenotypes, respectively. This means that a phenotype was average linked with about 2-3 phenotypes. Moreover, it was also found that phenotypes in each syndrome were almost the same, but slightly different (Wilcoxon rand-sum test). A validation computational method was presented to automatically determine a better MI-based association in the four algorithms.

### 3.3. Computational Validation Method of Established Networks

In order to automatically validate the different phenotype spectra discovered by the four algorithms, diagnosis information of the 2050 AP should be used. An AP subject included here was clinically diagnosed by at least three TCM experts to receive herbal treatment. The syndrome data was composed of seven syndromes. Name and frequency of syndromes are shown in Table 3 in a descending order. The data was represented by a 2050∗ 9 matrix, row represents a subject, and column represents a syndrome. If an AP subject is diagnosed as one of the seven syndromes, the corresponding cell of the matrix is denoted as 1, otherwise the cell is represented as 0.

In the supervised validation strategy, three computational measures (sensitivity, specificity, and accuracy) were employed to evaluate the coincidence of the four phenotype networks with the diagnosis information given by TCM experts. The algorithm was performed by the following three procedures.

**Procedure 1 1**

For each subnetwork (marked in different color) in the large phenotype network, it was returned to the phenotype data, if at least half phenotypes in the subnetwork simultaneously appear (their values are nonzero) on a subject, the serial number of the subject is recorded. The total number of each subnetwork was summed, denoted as  *M*.

**Procedure 2 2**

Tracking the serial number of a subnetwork to the syndrome data, a matrix with *M*∗  7 was retrieved.

**Procedure 3 3**

Three computational measures were calculated. The sensitivity is the ratio of the number of subjects diagnosed by the subnetwork to counterpart diagnosed by the TCM expert. The sensitivity describes the true positive of the subnetwork. The specificity refers to the ratio of the number of subjects not diagnosed by the subnetwork to the counterpart of the TCM experts. It describes the false negative of the subnetwork. The accuracy is the ratio of the number of subjects correctly (contains true positive and false negative) by the subnetwork to the counterpart of the TCM experts.

As given in Table 4, the supervised validation strategy-based association performed better than the other three algorithms. The average accuracy of the algorithm was higher than 80%, which means that the phenotype network coveys enough information of TCM clinical essence of AP. For a syndrome with high frequency in the context of AP, the algorithm achieved a high sensitivity. It obtained a high specificity for the syndrome with low frequency in AP. But the accuracy remains constantly, which indicated that the algorithm was not biased for any syndrome in AP.

### 3.4. Phenotype Networks for Combined Diseases

**Figure 3:**
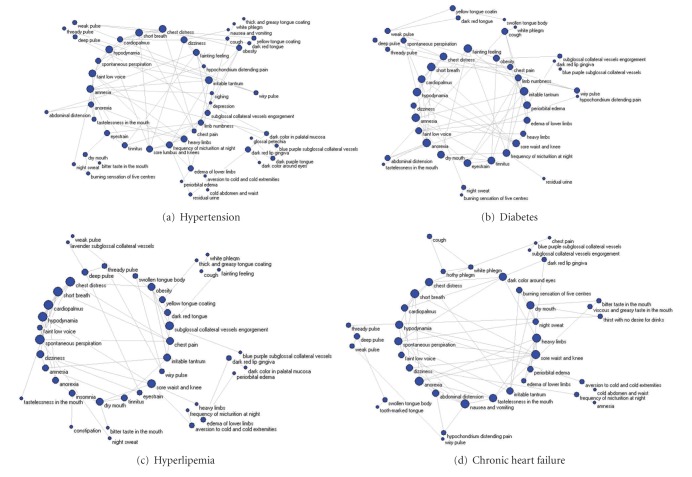
k-core phenotype of four CHD-combined diseases.

A parameter called degree of complex network was used to evaluate the phenotype networks for the four AP-combined diseases. A type of network called k-core network was used to build phenotype networks, from which different phenotype spectra among combined diseases were investigated. It was intuitively found in Figure 3 that four networks for AP combined with hypertension, diabetes, hyperlipemia, and chronic heart failure were different with each other, indicating that significant change of some phenotypes occurred in AP when combined with other diseases. In TCM theory, it means that syndromes in the context of combined diseases would significant change. Then, the treatment by Chinese herbals would accordingly change. The analysis of the difference between the four networks could guide the treatment of AP by TCM. It was found that when AP combined with hypertension the core syndromes were Blood stasis syndrome, Qi stagnation and hyperactivity of liver-Yang (or called excessive rising of liver-Yang). The last syndrome was absent from the whole network for AP (Figure 2(a)). While, in the network for diabetes, the phenotypes in the core network were hypodynamia, dizziness, tinnitus, frequency of micturition at night, tastelessness, and residual urine, which implied that Qi deficiency and Yin deficiency were core pathogenesis of AP combined with diabetes. The phenotype network in the AP combined with hyperlipemia, the core syndrome was found to be Tan-Zhuo with BSS. When AP was combined with chronic heart failure, the phenotypes turned to core syndrome with Yang deficiency with BSS. The variance in the phenotypes under the different combined diseases indicated an individual treatment strategy for AP.

## 4. Discussion and Conclusions

Accurate analysis of clinical syndromes is the premise of syndrome differentiation and treatment. In the clinical process of TCM, the large number and complexity, multilevel relationships of phenotypes had constrained the accuracy of syndrome differentiation. In our study, the MI method firstly described the association between phenotypes much more effective and without the intervention of subjective factors. The characteristics of phenotypes were in line with that of complex networks. Not only in common with special nature on the basis of their own evolutionary mechanisms, but also closely contacted with nature and structural features. Our research showed that MI and complex networks could be applied to the distribution rules study of phenotypes. In the phenotypes networks, we could explore the diagnostic rules of syndromes with core phenotypes or phenotype groups, analysis of basic syndromes of CHD patients and summarize the different syndromes of CHD patients with different comorbidities. In addition, researching the cores of the complex network means to find the “k-core network.” In k-core phenotype figures, the nodes for syndromes diagnosis had been showed clearly and intuitively. Combination of the degree values, the greater area that one node has, the more significant role it has played. In clinical diagnosis and treatment process, or during the epidemiological surveys, these core nodes (the core phenotypes) should be considered seriously.

In this paper, we did a clinical epidemiology of AP in CHD to collect 2050 subjects. Four revised mutual-information-based methods were presented to deeply understand the data, we take the positive occurrence frequency to rectify the inherent drawback of MI that prevents negative association from positive association pairs. It was found that revised MI could balance frequency and association and give a better measure of association between phenotypes. In the generation of complex phenotype network, we took a criterion that *P*_*A*_ and *P*_*B*_ composed a significant association pair if and only if *P*_*A*_ is one of the top *N* associated phenotypes of the phenotype *P*_*B*_ and vice versa. Compared to similar work with others that predefine the scale of the network, the algorithm proposed in this paper gives a more objective and convictive result. Pattern discovery based MI could achieve an accuracy of >80% with the diagnosis by TCM experts and discovered that there are seven syndromes considered as pathogenesis of CHD. By this algorithm and complex network analysis technique, it was found that the core pathogenesis of CHD combined with hypertension, diabetes, hyperlipemia, and chronic heart failure was Qi stagnation, Qi-Yin Deficiency, Tan-Zhuo, and Yang deficiency, respectively. The change in phenotype spectra when CHD was combined with other diseases provides a better insight into treating CHD by TCM with an individual way.

## Figures and Tables

**Table 1:** Basic statistics of 2050 cohort of AP.

	Frequency	Percentage
Male/female	1361/689	66.4%/33.6%
Hypertension	1374	67%
Diabetes	552	26.9%
Hyperlipemia	420	20.5%
Chronic heart failure	520	25.4%

**Table 2:** The top 10 phenotype pairs with largest revised mutual information in AP.

Phenotype pair		Revised mutual information
Chest distress*	Short breath*	0.29219
Periorbital edema	Edema of lower limbs	0.262114
Short breath*	Hypodynamia*	0.219433
Chest pain*	Chest distress*	0.219238
Cough	white phlegm	0.215073
Sighing	Depression	0.202779
Short breath*	Cardiopalmus*	0.190918
Amnesia	dizziness	0.181577
Anorexia	Tastelessness in the mouth	0.158838
Chest distress*	Cardiopalmus*	0.14342

**Table 3:** The frequency of diagnosed seven syndromes in the context of AP.

Syndrome	Frequency	Syndrome	Frequency	Syndrome	Frequency
Qi deficiency syndrome	1409/2050 (68.73%)	Tan-Zhuo syndrome	696/2050 (33.95%)	Spleen deficiency syndrome	210/2050 (10.24%)
Blood stasis syndrome	1375/2050 (67.07%)	Yang deficiency syndrome	391/2050 (19.07%)	—	—
Yin deficiency syndrome	775/2050 (37.80%)	Qi stagnation Syndrome	236/2050 (11.51%)	—	—

**Table 4:** The computational performance of the four MI-based algorithms.

Syndrome	Algorithm	Sensitivity	Specificity	Accuracy
Qi deficiency syndrome	1	0.911497105	0.634958383	0.79804878
	2	0.819699499	0.498826291	0.686341463
	3	0.829592685	0.514757969	0.699512195
	4	0.804898649	0.473441109	0.664878049

Blood stasis syndrome	1	0.8408	0.595	0.744878049
	2	0.909171861	0.618122977	0.777560976
	3	0.828371278	0.52753304	0.695121951
	4	0.900179856	0.601279318	0.763414634

Yin deficiency syndrome	1	0.843273232	0.87434161	0.863414634
	2	0.80112835	0.845637584	0.830243902
	3	0.773049645	0.828996283	0.809756098
	4	0.812849162	0.855322339	0.840487805

Tan-Zhuo syndrome	1	0.806451613	0.877769836	0.855121951
	2	0.781701445	0.853538893	0.831707317
	3	0.806299213	0.869964664	0.850243902
	4	0.793333333	0.848275862	0.832195122

Yang deficiency syndrome	1	0.724233983	0.922531047	0.887804878
	2	0.710144928	0.914369501	0.88
	3	0.630985915	0.890962099	0.854634146
	4	0.690625	0.901734104	0.868780488

Qi stagnation syndrome	1	0.707964602	0.958333333	0.930731707
	2	0.7	0.948108108	0.923902439
	3	0.731707317	0.953387534	0.931219512
	4	0.641025641	0.940161725	0.911707317

Spleen deficiency syndrome	1	0.757575758	0.967602592	0.947317073
	2	0.773333333	0.950526316	0.937560976
	3	0.752808989	0.959401709	0.941463415
	4	0.703703704	0.949152542	0.929756098

## References

[1] World Health Organization (1994). *World Health Statistics Annual, 1993*.

[2] Wu X, Jiang R, Zhang MQ, Li S (2008). Network-based global inference of human disease genes. *Molecular Systems Biology*.

[3] Li S, Zhang Z, Wu L, Zhang X, Li Y, Wang YY (2007). Understanding ZHENG in traditional Chinese medicine in the context of neuro-endocrine-immune network. *IET Systems Biology*.

[4] Matsumoto C, Kojima T, Ogawa K (2008). A proteomic approach for the diagnosis of “Oketsu” (blood stasis), a pathophysiologic concept of Japanese traditional (Kampo) medicine. *Evidence-Based Complementary and Alternative Medicine*.

[5] Guo S, Chen JX, Zhao HH (2009). Building and evaluating an animal model for syndrome in traditional Chinese medicine in the context of unstable angina (Myocardial Ischemia) by supervised data mining approaches. *Journal of Biological Systems*.

[6] Liu Y, Yin HJ, Chen KJ (2011). Research on the correlation between platelet gelsolin and blood-stasis syndrome of coronary heart disease. *Chinese Journal of Integrative Medicine*.

[7] Wang WR, Lin R, Zhang H (2011). The effects of Buyang Huanwu Decoction on hemorheological disorders and energy metabolism in rats with coronary heart disease. *Journal of Ethnopharmacology*.

[8] Gibbons RJ, Chatterjee K, Daley J (1999). ACC/AHA/ACP-ASIM guidelines for the management of patients with chronic stable angina: a report of the American College of Cardiology/American Heart Association Task Force on Practice Guidelines (Committee on Management of Patients With Chronic Stable Angina). *Journal of the American College of Cardiology*.

[9] Chen J, Xi G, Chen J (2007). An unsupervised pattern (syndrome in traditional Chinese medicine) discovery algorithm based on association delineated by revised mutual information in chronic renal failure data. *Journal of Biological Systems*.

[10] Phillip B (2008). Exploratory social network analysis with pajek. *Sociological Methods Research*.

[11] Li S, Zhang B, Jiang D, Wei YY, Zhang NB (2010). Herb network construction and co-module analysis for uncovering the combination rule of traditional Chinese herbal formulae. *BMC Bioinformatics*.

